# Gains in cognition through combined cognitive and physical training: the role of training dosage and severity of neurocognitive disorder

**DOI:** 10.3389/fnagi.2015.00152

**Published:** 2015-08-07

**Authors:** Panagiotis D. Bamidis, Patrick Fissler, Sokratis G. Papageorgiou, Vasiliki Zilidou, Evdokimos I. Konstantinidis, Antonis S. Billis, Evangelia Romanopoulou, Maria Karagianni, Ion Beratis, Angeliki Tsapanou, Georgia Tsilikopoulou, Eirini Grigoriadou, Aristea Ladas, Athina Kyrillidou, Anthoula Tsolaki, Christos Frantzidis, Efstathios Sidiropoulos, Anastasios Siountas, Stavroula Matsi, John Papatriantafyllou, Eleni Margioti, Aspasia Nika, Winfried Schlee, Thomas Elbert, Magda Tsolaki, Ana B. Vivas, Iris-Tatjana Kolassa

**Affiliations:** ^1^Laboratory of Medical Physics, Faculty of Health Sciences, Medical School, Aristotle University of ThessalonikiThessaloniki, Greece; ^2^Institute of Psychology and Pedagogy, Clinical and Biological Psychology, Ulm University, UlmGermany; ^3^Behavioral Neurology and Neuropsychology Unit, 1st and 2nd Department of Neurology, Medical School, National Kapodistrian University of AthensAthens, Greece; ^4^Greek Association of Alzheimer’s Disease and Related Disorders, ThessalonikiGreece; ^5^Cognitive Psychology and Neuropsychology Lab, Department of Psychology, City College, The University of Sheffield International Faculty, ThessalonikiGreece; ^6^Department of Psychiatry and Psychotherapy, University of RegensburgRegensburg, Germany; ^7^Clinical Psychology and Clinical Neuropsychology, University of KonstanzKonstanz, Germany; ^8^3rd Department of Neurology, Medical School, Aristotle University of ThessalonikiThessaloniki, Greece

**Keywords:** physical training, cognitive training, combined intervention, exergames, mild cognitive impairment, dementia, neurocognitive disorder, aging

## Abstract

Physical as well as cognitive training interventions improve specific cognitive functions but effects barely generalize on global cognition. Combined physical and cognitive training may overcome this shortcoming as physical training may facilitate the neuroplastic potential which, in turn, may be guided by cognitive training. This study aimed at investigating the benefits of combined training on global cognition while assessing the effect of training dosage and exploring the role of several potential effect modifiers. In this multi-center study, 322 older adults with or without neurocognitive disorders (NCDs) were allocated to a computerized, game-based, combined physical and cognitive training group (*n* = 237) or a passive control group (*n* = 85). Training group participants were allocated to different training dosages ranging from 24 to 110 potential sessions. In a pre-post-test design, global cognition was assessed by averaging standardized performance in working memory, episodic memory and executive function tests. The intervention group increased in global cognition compared to the control group, *p* = 0.002, Cohen’s *d* = 0.31. Exploratory analysis revealed a trend for less benefits in participants with more severe NCD, *p* = 0.08 (cognitively healthy: *d* = 0.54; mild cognitive impairment: *d* = 0.19; dementia: *d* = 0.04). In participants without dementia, we found a dose-response effect of the potential number and of the completed number of training sessions on global cognition, *p* = 0.008 and *p* = 0.04, respectively. The results indicate that combined physical and cognitive training improves global cognition in a dose-responsive manner but these benefits may be less pronounced in older adults with more severe NCD. The long-lasting impact of combined training on the incidence and trajectory of NCDs in relation to its severity should be assessed in future long-term trials.

## Introduction

As a result of the population aging, dementia affects a growing number of individuals (Alzheimer’s Association, 2014). Next to the rising emotional toll of dementia, the financial costs are expected to more than double in the upcoming 30 years (Hurd et al., 2013). As pharmacological treatment show limited clinical effects on cognition (Schneider et al., 2014), behavioral approaches aiming to promote cognitive performance become increasingly important (Imtiaz et al., 2014). Single component cognitive and physical training improved specific cognitive functions (Kramer et al., 1999; Ball et al., 2002). However, (1) inconsistent and limited generalizing benefits on global cognition were found (see, e.g., Kelly et al., 2014b; Rebok et al., 2014), (2) effect modifiers of training-induced effects such as severity of neurocognitive disorder (NCD), age, or gender are largely unexplored (Leckie et al., 2012; Walton et al., 2014), (3) the impact of training dosage is still unclear (see Liu-Ambrose et al., 2010; Ball et al., 2013 for rare dose-response studies), and (4) current findings have limited generalizability to potential end users as most studies applied highly restricted selection criteria including only sedentary or healthy participants (see, e.g., Smith et al., 2009; Erickson et al., 2011). This study aims to overcome these four shortcomings by using a combined physical and cognitive training intervention in a community-dwelling sample of potential end users with and without NCD while manipulating training dosage and investigating effect-modifying effects in an exploratory approach.

Cognitive as well as physical training interventions have been shown to enhance performance in untrained cognitive tasks (see Hindin and Zelinski, 2012 for a meta-analysis). However, both approaches have their limitations. Cognitive training induced only limited transfer effects, i.e., cognitive training improved performance in untrained cognitive tasks which were structurally very similar to the training tasks (Rebok et al., 2014) but showed no (Ball et al., 2002; Owen et al., 2010; Chacko et al., 2014) or only limited transfer effects to structurally dissimilar tasks (Harrison et al., 2013). Especially in older adults, in contrast to younger adults, far-transfer effects to structurally dissimilar tasks could not be found (Schmiedek et al., 2010). Some cognitive training programs revealed effects on untrained, structurally rather dissimilar tasks, but they improved only specific functions such as memory (Barnes et al., 2009; Zelinski et al., 2011) rather than global cognition (but see also Lampit et al., 2014a).

Physical training interventions such as resistant and aerobic training have shown benefits on tasks of specific cognitive functions (e.g., Kramer et al., 1999; Lautenschlager et al., 2008; Liu-Ambrose et al., 2010). However, different meta-analyses and systematic reviews did not come to univocal conclusions about cognitive benefits (see Colcombe and Kramer, 2003; Angevaren et al., 2008; van Uffelen et al., 2008; Smith et al., 2010b; Kelly et al., 2014b for reviews and meta-analysis). While an older meta-analysis showed large and specific benefits on executive function (Colcombe and Kramer, 2003), a more recent meta-analysis revealed small benefits on several functions (Hedges’ g < 0.16, Smith et al., 2010b). The most recent meta-analysis by Kelly et al. (2014b) found no significant cognitive benefit of aerobic training and very function-specific benefits of resistance training. Taking all results together, it seems that cognitive benefits of physical training interventions are very small-sized and by their own not of practical significance after short-term interventions.

How can we overcome the limitations of mono-therapeutical approaches? As cognitive decline is multi-causal (see, e.g., Buckner, 2004), multi-component interventions acting by multiple mechanisms may be necessary for practically significant effects on global cognition (Ngandu et al., 2015). Physical and cognitive trainings act by different mechanisms on cognition. Some mechanisms may potentiate each other (i.e., synergistic effects) while others may merely add up (see Kempermann, 2008; Fabel et al., 2009; Kraft, 2012; Fissler et al., 2013; Hötting and Röder, 2013; Bamidis et al., 2014 discussing this issue).

Synergistic effects of both interventions may arise by a “plasticity facilitation” effect of physical training which, in turn, is “guided” by cognitive training to induce its beneficial cognitive effect. According to the so-called “guided plasticity facilitation” framework by Fissler et al. (2013), physical training facilitates synaptic plasticity and neurogenesis via growth factors such as brain-derived neurotrophic factors and insulin-like growth factor-1 (see, e.g., Cotman et al., 2007). Cognitive training, in turn, “guides” the facilitated plastic potential by regulating synapse formation and elimination (cf. Trachtenberg et al., 2002), as well as by enhancing the survival of physical training-induced newborn cells (Fabel et al., 2009). Thus, combined physical and cognitive training may potentiate their impact to restructure neuronal networks, resulting in enhanced processing efficiency (Subramaniam et al., 2014).

Training types may also act by additive and independent mechanisms on cognition (Wolf et al., 2006). Physical training may reduce neuroinflammation (Cotman et al., 2007), increase cerebral blood flow (Smith et al., 2010a) and velocity (Ainslie et al., 2008), decrease risk factors for cognitive decline such as cardiovascular diseases and diabetes (Cotman et al., 2007), reduce amyloid deposition (Liang et al., 2010) and increase hippocampal size (Erickson et al., 2011). Cognitive training may reduce the impairment of hippocampal long-term potentiation induced by amyloid-β oligomers (Li et al., 2013) and may reduce amyloid deposition independently from physical training (Lazarov et al., 2005; Landau et al., 2012).

What is the empirical evidence for the efficacy of combined physical and cognitive training interventions? Recent findings indicate beneficial effects of combined training on cognitive functions (Fissler et al., 2013; Law et al., 2014; Ngandu et al., 2015) and some studies indicate more benefits through combined training than through each component alone (Fabre et al., 2002; Oswald et al., 2006; Shah et al., 2014). Also an animal study found that combined training yielded more cognitive benefits than each component by its own (Langdon and Corbett, 2012). However, “research to assess the impact of combined cognitive and physical training on cognitive functions in older adults is still in its fledgling stage” (Law et al., 2014).

A huge and heterogeneous set of cognitive and physical training programs is currently available. Technology assisted solutions engaging the elderly in physical training through gaming have been increasingly investigated in recent years and the term “exergaming” has even been coined to describe this notion (Robert et al., 2014). However, in contrast to currently available exergames, we developed a service which is tailor-made for elderly use and integrates both physical and cognitive game-like trainings under a unified user interface powered by web service technologies (Konstantinidis et al., 2010; Bamidis et al., 2011). Programs with the most robust empirical evidence for transfer effects on cognitive functions in older adults were implemented in this system. A Greek version of a well-validated neuroplasticity-based training program (*Brain Fitness Program;* Posit Science Corporation, San Francisco, CA, USA) was used as the cognitive training component (Mahncke et al., 2006a). This program improved performance in verbal memory tasks that are structurally rather dissimilar from the training tasks (Smith et al., 2009; Zelinski et al., 2011). It targets auditory processes as well as working memory processes. The physical training program included both resistance and aerobic training, as their combination seems to be most effective (Colcombe and Kramer, 2003; Kelly et al., 2014b). Additional balance and flexibility exergames were designed and implemented to meet the needs of elderly users (Konstantinidis et al., 2014).

To address the lack of knowledge with respect to effect modifiers of cognitive (Walton et al., 2014) and physical training (Leckie et al., 2012), we conducted an exploratory analysis regarding the potential impact of severity of NCD, baseline cognitive performance, education, age, gender, and social activity level on the intervention effect.

Previous studies of physical and cognitive training could not clarify the impact of training dosage on cognitive improvement (see Liu-Ambrose et al., 2010 for rare studies investigating training dosage; Ball et al., 2013). A dose-response effect strengthens evidence for a causal role of the intervention components (Hill, 1965). Moreover, dose-response effects have considerable practical relevance. Guidelines and recommendations for end users can be derived (Robert et al., 2014). In this study, we thus investigated the effect of training dosage on cognitive benefits.

Lastly, the generalizability of previous findings to potential end users was restricted as often strict selection criteria were applied. These criteria included a sedentary lifestyle (e.g., Erickson et al., 2011) or no neurocognitive and psychiatric disorders (e.g., Smith et al., 2009). To overcome this limitation, we used unrestrictive criteria, not excluding older adults with an active lifestyle, participants with mild cognitive impairment (MCI), dementia and psychiatric disorders, if the conditions did not preclude participation in the intervention.

Taken together, we hypothesized that combined cognitive and physical training improves global cognition in contrast to a passive control group and that the number of completed training sessions predicts cognitive benefits. In addition, we explored potential effect modifiers of training-induced cognitive benefits.

## Materials and Methods

### Design

The multi-center study was part of the Long Lasting Memories (LLM) project (http://www.longlastingmemories.eu), which was funded by the European Commission [Information and Communication Technologies Policy Support Program (ICT-PSP)] for a 3 years period (2009–2012). The trial was registered retrospectively in ClinicalTrials.gov (Identifier: NCT02267499).

We used a pre-post-test design and allocated participants to the passive control group and the intervention group. Intervention group participants were allocated to different training dosages ranging from 24 to 110 potential sessions (*M* = 59; SD = 21). This large-scale computerized intervention study with different training dosages did not allow randomized allocation due to feasibility and practical issues as well as due to time and financial limitations of the project. However, both allocation to group (training vs. passive controls) and to training dosage was driven by non-systematic practical and logistic reasons (such as the timing of the next start of training or the time period until the next national holidays or the number of successfully screened and pretested participants at a given point in time) and was not influenced by participant’s choice, motivation or compliance. We cannot exclude a potential bias through this allocation procedure but we are not aware of a mechanism which biased results favoring the intervention group or favoring a higher training dosage.

Post-test was conducted within 2 weeks after completion of the training period. The interventions reported in this paper were carried out in Athens and Thessaloniki (Greece) within day care centers, hospitals, senior care centers, a memory outpatient center, local parishes, at university campus facilities (university community installations), and at participant’s homes (Bamidis, 2012; Billis et al., 2013).

Severity of NCD, baseline cognitive performance, education, age, gender, and social activity level were used as potential effect modifiers of training effects. Global cognition served as the primary outcome and cognitive functions such as episodic memory, working memory, and executive function were defined as secondary outcomes.

### Participants

The study enrolled 322 community-dwelling older adults ranging from cognitively healthy individuals to individuals with MCI or dementia [Mini Mental State Examination score (MMSE) 18–30]. According to a power analysis, more participants had to be allocated to the intervention group than the control group to achieve the same power in the dose-response analysis and in the group analysis. Our study had more than 95% power to detect a medium effect size in the dose-response analysis (*r* = 0.3) and the group analysis (*f* = 0.25) assuming two-tailed testing with a significance level of α = 0.05.

Inclusion criteria were age ≥55 years, no severe cognitive impairment (MMSE ≥ 18; cf. Tombaugh and McIntyre, 1992), fluent language skills, agreement of a medical doctor and time commitment to the test and training protocol. Exclusion criteria were concurrent participation in another study, severe physical or psychological disorders which precluded participation in the intervention (i.e., inability to follow instructions), unrecovered neurological disorders such as stroke, traumatic brain injury, unstable medication within the past 3 months, severe and uncorrectable vision problems, or hearing aid for less than 3 months. As there were only three participants with Parkinson’s disease in the intervention group and none in the control group, these were excluded from the data analysis.

Recruitment strategies included flyers, workshops, presentations, and professional contacts in the intervention and associated institutions, advertisement in the local newspapers, and word of mouth. Participants received no compensation; the training program was provided at no cost.

The protocol was approved by the Bioethics Committees of two Medical Schools, the Medical School of the National and Kapodistrian University of Athens and the Medical School of the Aristotle University of Thessaloniki, as well as, the Board of the Greek Association of Alzheimer’s Disease and Related Disorders. Participants provided written informed consent prior to study participation.

### Intervention

The computerized training program was conducted by using an integrated web-service system composed of a physical as well as a cognitive training component through a universal interface, facilitated by touch screen systems (Konstantinidis et al., 2010; Bamidis et al., 2011). It was carried out in a group setting apart from one participant who used the training system at home.

#### Physical Training

The computerized physical training program *FitForAll* (llmcare.gr/el/service/fitforall, Billis et al., 2010; Konstantinidis et al., 2014) was composed of (1) aerobic, (2) strength, (3) balance and (4) flexibility trainings and exergames. Physiotherapists, sport experts/physical educators, psychologists, or trained facilitators (formal care givers) introduced participants to the training program and consulted participants with respect to the training intensity level. A 10-min warm-up phase preceded the four different training components (10–15 min each), followed by a 5-min cool-down phase. Participants started on the light intensity level with a target heart rate (HR) of 50–60% of maximum heart rate (HR_max_) and could proceed to the very hard level with a target HR of 80–90% of HR_max_. Training was embedded in game-like tasks using either the *Wii Balance Board* or the *Wii Remote* which measure the center of mass and limb movements, respectively. (1) The *FitForAll* exergames “Hiking” and “Cycling” are two aerobic trainings in which participants run on the spot or cycle on a stationary mini-bike, thereby moving the bicycle of an avatar through a city landscape. (2) Training tasks aiming to increase upper and lower limb strength consisted of weightlifting and resistance trainings. Pictures of positive valence were revealed gradually with increasing repetitions. (3) “Ski Jump” is a static balance task asking participants to move their center of mass to a specific position, thus controlling the avatar’s jump performance. “Arkanoid*”* is designed to train dynamic balance. Participants needed to control the horizontal position of a bar aiming to hit a moving ball which, in turn, needed to be directed to destroy bricks. In “Apple Tree,” participants practiced dynamic balance by controlling a basket which served to pick apples from a tree. “Fishing” is a dynamic balance game in which participants needed to control the vertical position of a boat with the goal to fish horizontally moving fishes. In “Golf” participants moved a ball around barriers into a hole using their center of mass. (4) Flexibility training consisted of stretching and warm-up trainings.

#### Cognitive Training

A localized version (adapted in terms of Greek language and cultural contexts) of the *Brain Fitness Program* (Posit Science Corporation, San Francisco, CA, USA, see Mahncke et al., 2006b) served as the cognitive training component (Bamidis, 2012). It consisted of six tasks targeting auditory processing and working memory. With task progression, increasingly long arrays of syllables up to words, sentences and narratives were used. The stimuli were synthetically processed, enabling variations in duration and amplitude of rapid frequency modulations within sounds and speech to adapt difficulty. The program presented, via head-phones, difficult-to-discriminate auditory stimuli which were partly interwoven in tasks with high working memory load. Two tasks were psychophysical auditory training tasks (“High or Low*”* and “Tell us Apart”), while three tasks tapped both working memory and auditory processing (“Sound Replay,” “Listen and Do,” “Match It”). In “Story Teller,” stories with increasing demands on auditory perception were presented and participants subsequently needed to recognize story facts out of multiple possible answers. Feedback was given by rewarding correct responses with points while gradually revealing background pictures of positive valence. Difficulty level was continuously adapted based on participants’ performance. Psychologists introduced participants to the training program and consulted participants with respect to the training intensity level.

### Measures

#### Cognitive Outcomes

Greek versions of the *California Verbal Learning Test* (Delis et al., 1987), the *Digit Span Test* (Wechsler, 1997), and the *Trail Making Test* (TMT, Reitan, 1958) were used to assess cognitive outcomes. Measures are well-validated (English versions; Sanchez-Cubillo et al., 2009; Beck et al., 2014) and possess good reliability (retest-reliability in the control group of this study for global cognition was good; *r*_pre-post_ = 0.82; on average, 67 days between tests). All measures are widely used in clinical practice and comprise a wide spectrum of cognitive functions affected in normal aging (Park et al., 2002), MCI (Economou et al., 2007), and dementia (American Psychiatric Association, 2013). In the verbal learning test, five learning trials of an orally presented 16-word shopping list (list A) were followed by an interference shopping list (list B) as well a short-delayed recall of list A with and without category cues. After another 20 min, participants were asked to recall list A with and without category cues. In the Forward and Backward Digit Span Test participants were asked to repeat an increasingly long sequence of orally presented digits in same and in reverse order of presentation. In the TMT part A, participants needed to draw a line between numbers in ascending order. In part B, numbers and letters needed to be connected in alternating alphabetic and ascending orders. The difference of time needed to complete part B and part A (TMT B-A) is suggested to be a measure of the switching component of executive function (Sanchez-Cubillo et al., 2009). If part A lasted longer than 3 min and part B lasted longer than 5 min, the test was stopped and coded with the maximum time of 180 or 300 s, respectively. In case one subtest was not completed within time, TMT B-A could not be calculated. This affected 29% of the intervention group and 26% of the control group.

##### Specific cognitive functions and global cognition

Secondary outcome measures including episodic memory, working memory, and executive function were calculated by averaging *z*-standardized sub-scores of the three cognitive tests. For episodic memory sub-scores comprised the total number of recalled words within the five learning trials and the 20-min free delayed recall score. For working memory the sub-scores comprised the Digit Span Forward and the Digit Span Backward Test. Executive function was calculated by inverting *z*-standardized TMT B-A scores. The primary outcome measure, global cognition, was a composite score derived from all three cognitive function scores, calculated by averaging the *z*-standardized scores of episodic memory, working memory, and executive function. Baseline assessment served for *z*-standardization (score minus baseline mean divided by baseline standard deviation). Global cognition was calculated if at least two of three cognitive function scores were available for analysis.

#### Psychological, Physical, and Daily Living Outcomes

Quality of life was assessed with the short-version of the World Health Organization Quality of Life questionnaire (WHOQOL-BREF, Skevington et al., 2004), measuring physical, psychological, social, and environmental domains. Depressive symptoms were assessed with the short, 15-item version of the Geriatric Depression Scale (GDS-short, Sheikh and Yesavage, 1986). Daily life functioning was assessed with the Instrumental Activities of Daily Living Scale (IADL, Lawton and Brody, 1969). Physical fitness was operationalized with the composite score of the averaged *z*-standardized subtests of the Senior Fitness Test (Chair stand, Arm curl, 2-min step, Back scratch, Chair sit-and-reach, 8-foot up-and-go, Rikli and Jones, 2001). This measure was collected only in a subsample (*n* = 119; intervention group, *n* = 84; control group, *n* = 35). Greek versions (validated or adapted for research) of all tests were used.

#### Moderator Variables and Group Characteristics

An interview served to collect demographic data such as education, age, gender, and medical data. The Mini Mental State Examination was used as a cognitive screening test (MMSE, Folstein et al., 1975). NCDs were assessed by neurologists on the basis of a clinical interview with the patient and an informant, clinical examination including neurobehavioral examination and, if available, imaging (CT or MRI) and standard blood and biochemistry investigations according to the EFNS-ENS guidelines (Waldemar et al., 2000; Sorbi et al., 2012) and AAN practice parameters for differential diagnosis of dementia (Knopman et al., 2001; Pitner and Bachman, 2004). Diagnosis was made in accordance with the DSM-IV and ICD-10 criteria for dementia and Petersen’s criteria for MCI (Petersen, 2004). All individuals with MCI had a Clinical Dementia Rating (Hughes et al., 1982) score of 0.5. To assess NCD as a moderator of training effects it was treated as an ordinally scaled variable with the values “healthy” < “MCI” < “dementia.” The number of social activities including sport activities, church activities, volunteer work, meetings for seniors, club meetings, and other social activities served as a measure of the social activity level. In case of missing values for one kind of social activity, the value was estimated by the mean score of the other social activities. Training dosage was operationalized by the total number of completed cognitive and physical training sessions which were collected electronically via online data records and web services (Bamidis et al., 2011).

### Data Analysis

Statistical analysis was conducted using the R statistical software package version 2.15.1 (R Development Core Team, 2011). Baseline group characteristics were compared using *t*-tests for continuous variables and χ^2^-tests for categorical variables.

To assess the intervention effect, multiple regression models were used as the primary analysis. Change in cognitive performance was the dependent variable. Covariates were included in the primary analysis to enhance statistical power through the reduction of variance in the dependent variable which was attributable to other factors than the intervention. Study center (dummy-coded; Thessaloniki vs. Athens) was included according to established procedures in multi-center studies (Kahan and Morris, 2013), accounting for similarities of participant’s within centers and differences between center characteristics. Selection of other predictors was based on the forward and backward Akaike Information Criterion (AIC)-stepwise regression. Baseline performance, age and education reduced the AIC and were selected as covariates. The difference in performance change between intervention and control group was assessed by adding group (dummy-coded) to the model.

An available-case analysis – consistent with the modified intention-to-treat approach of randomized controlled trials – was conducted: all participants with available outcomes were included according to the originally allocated group, irrespective of any consideration such as the initiation and completion of the designated intervention. Imputation methods for missing data were not used as the strong assumptions required by these methods cannot be justified and violation of assumptions induce an estimation bias (Streiner, 2008). Analyzing all participants according to the initial group assignment irrespective of the intervention received, reduces self-selection and the risk of an attrition bias (Flick, 1988). In contrast with a per-protocol analysis, non-compliance with the allocated treatment is ignored, thus depicting a more conservative analysis, which tends to underestimates the true effect size of the treatment (Moher et al., 2010).

To assess the robustness of group effects, we conducted a secondary analysis without accounting for other variables (see Supplementary Table S1). This method yields the same results as the Group [intervention vs. control] × Session [pre vs. post] interaction using repeated-measure ANOVA or linear mixed effect models (Pinheiro et al., 2010; see Supplementary Table S1).

To assess moderator effects (i.e., effect modifiers), an interaction term between each moderator variable and group was added separately as predictor. As we tested six moderator variables, we report both unadjusted *p*-values and *p*-values adjusted for six multiple comparisons by using Holm’s method (Holm, 1979). In this exploratory analysis which aims for hypothesis generation rather than rigorous hypothesis testing, *p*-value adjustment is not viewed as necessary (Rothman, 1990; Roback and Askins, 2005). However, results should be cautiously interpreted as the risk of false positives increases with multiple testing.

Multiple regression models within exercising participants served to evaluate the effect of training dosage on change in cognitive performance. These models included the number of completed training sessions and the covariates as predictor variables of performance change.

To calculate effect sizes all outcome measures were *z*-standardized according to the baseline data of both groups. Cohen’s *d* represents the estimated *z*-standardized difference between the change in the intervention group and the change in the control group, accounting for the covariates. Statistical significance tests were two-tailed with a significance level of α = 0.05.

## Results

### Baseline Group Characteristics

**FIGURE 1:**
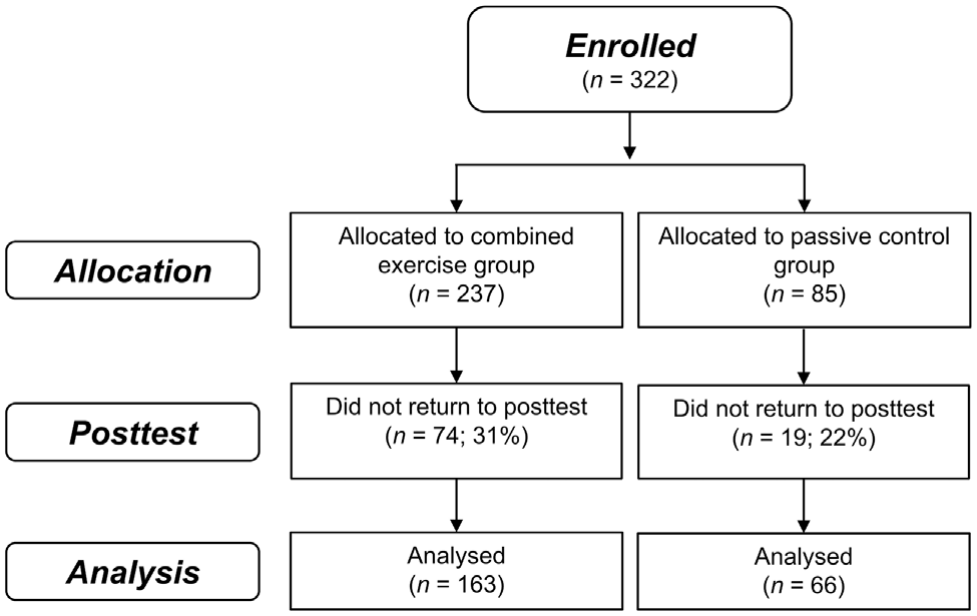
**Flow of participant chart.** Flow of participants within the intervention and passive control group.

A total of 322 participants were enrolled in the study from June 22, 2010 (intervention group, *n* = 237; passive control group, *n* = 85; Thessaloniki, *n* = 177; Athens, *n* = 145), 229 completed the post-test until April 04, 2012 (intervention group, *n* = 163; passive control group, *n* = 66; Thessaloniki, *n* = 120; Athens, *n* = 109). Attrition rates were 31% in the intervention group and 22% in the control group which were not significantly different, χ^2^(1) = 1.98; *p* = 0.16 (see **Figure 1**). On average, participants of the intervention group completed 37 (SD = 19.8) training sessions (23 cognitive and 14 physical) within an average period of 6-weeks. Baseline characteristics are depicted in **Table 1**. Apart from significantly more depressive symptoms in the intervention group (*M* = 2.8, SD = 2.7) compared to the control group [*M* = 2.0, SD = 2.0, *t*(225) = 2.08; *p* = 0.04], there were no other significant group differences, *p*s ≥ 0.05 (see **Table 1**). The group difference in the quality of life questionnaire WHOQOL-BREF was marginally significant, *p* = 0.05.

**Table 1:** Baseline characteristics of intervention group and passive control group.

Measure	Intervention group (*n* = 163)	Control group (*n* = 66)	*p*-value^a^
Demographic data			
Age, mean ± SD	71.3 ± 7.1	70.1 ± 8.1	0.25
Female, *n* (%)	117/162 (72%)	41/66 (62%)	0.18
Education, years mean ± SD	10.9 ± 4.9	10.7 ± 4.4	0.77
Cognitive data			
MMSE, mean ± SD	26.8 ± 2.9	26.4 ± 2.9	0.29
Global cognition, mean ± SD	0.0 ± 1.0	-0.1 ± 1.0	0.43
Cognitive diagnosis			0.12
Healthy, n/n_group_ (%)	69/163 (42%)	21/66 (32%)	
MCI, n/n_group_ (%)	72/163 (44%)	39/66 (59%)	
Dementia, n/n_group_ (%)	22/163 (13%)	6/66 (9%)	
Psychological data			
GDS-short, mean ± SD	2.8 ± 2.7	2.0 ± 2.0	0.04
WHOQOL-BREF composite, mean ± SD	-0.1 ± 1.0	0.2 ± 1.0	0.05
Medical data			
No. of medications, mean ± SD	3.4 ± 2.3	2.8 ± 2.5	0.17
Diabetes mellitus, n/n_group_ (%)	21/154 (14%)	3/55 (5%)	0.17
Hypertension, n/n_group_ (%)	76/154 (49%)	21/55 (38%)	0.20
High cholesterol, n/n_group_ (%)	34/153 (22%)	15/55 (27%)	0.57
Currently smoking, n/n_group_ (%)	18/155 (12%)	9/56 (16%)	0.53
Social data			
Number of social activities, mean ± SD	2.2 ± 1.0	2.5 ± 1.3	0.16
Number of children, mean ± SD	1.8 ± 0.9	1.9 ± 0.7	0.57
Living alone, n/n_group_ (%)	48/161 (30%)	11/60 (18%)	0.12
Study data			
Total training sessions, mean ± SD	37.3 ± 19.9	–	–
Physical training sessions, mean ± SD	14.5 ± 11.2	–	–
Cognitive training sessions, mean ± SD	22.8 ± 10.0	–	–
Trial site, n/n_group_ (%) of Thessaloniki	88/163 (54%)	32/66 (48%)	0.54
Days between pre- and post-test, mean ± SD	64.4 ± 30.0	67.4 ± 45.9	0.57
Attrition rates, n/n_group_ (%)	74/237 (31%)	19/85 (22%)	0.16

*^a^*p-*values of group comparisons refer to *t*-tests for continuous variables and to χ^2^ tests for categorical variables.*

*WHOQOL-BREF, short version of the World Health Organization Quality of Life questionnaire; MMSE, Mini Mental State Examination; MCI, mild cognitive impairment.*

### Does Combined Training Improve Global Cognition?

**FIGURE 2:**
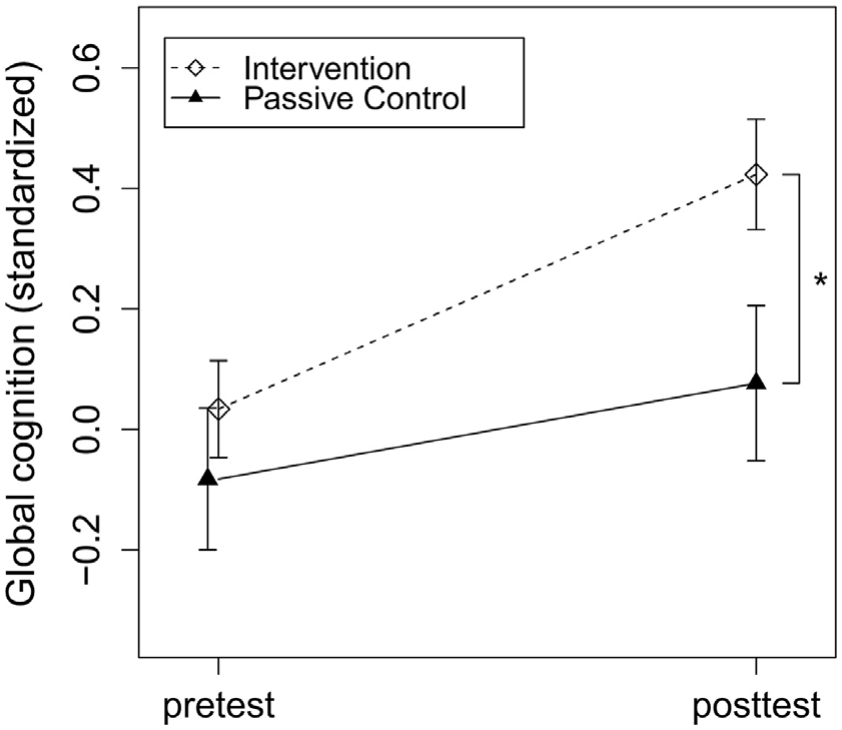
**Intervention effects on global cognition.** Intervention and passive control group comparison of *z*-standardized pre- and post-test global cognition. The asterisk indicates a significant beneficial effect of the intervention compared to the control group on post-test performance accounted for baseline cognitive performance, education, age and study center, *p* = 0.002, Cohen’s *d* = 0.31. Arrows represent SE.

To assess intervention effects, the dummy-coded variable group (intervention vs. control group) was added to the regression model accounting for baseline cognitive performance, education, age, and study center. In accordance with our hypothesis, the intervention group compared to the control group significantly improved in global cognition, *t*(219) = 3.20, *p* = 0.002, Cohen’s *d* = 0.31 (see **Figure 2**). Regarding secondary outcomes, the intervention group compared to the control group significantly improved in executive function, *t*(156) = 2.56, *p* = 0.01, Cohen’s *d* = 0.37, and episodic memory, *t*(216) = 2.21, *p* = 0.03, Cohen’s *d* = 0.20. There was no significant effect of group on change in working memory, *t*(219) = 1.29, *p* = 0.20, Cohen’s *d* = 0.15 (see **Table 1**).

Previous studies about the same cognitive training program found near-transfer effects on verbal working memory in cognitively healthy participants (Mahncke et al., 2006b; Smith et al., 2009; Zelinski et al., 2011), but not in participants with probable MCI (Barnes et al., 2009, 2013). Therefore, we performed a subgroup analysis of cognitive training effects in cognitively healthy participants. Consistent with previous finding, a significant effect of group was found, *t*(83) = 2.19, *p* = 0.03, Cohen’s *d* = 0.42.

As depressive symptoms differed significantly between groups, we accounted for this variable in an additional analysis. Results did not change. Using the secondary method of analysis, which did not account for covariates, revealed consistent results, apart from a non-significant effect in episodic memory (see Supplementary Table S1), indicating that effects on global cognition and executive function are most robust.

### Do Cognitive Benefits Depend on Individual Differences?

To explore modifying variables of training effects, we added group, the respective moderator variable and an interaction term of both variables to the regression model accounting for baseline cognitive performance, education, age, and study center. The ordinally coded variable severity of NCD (healthy < MCI < dementia), baseline cognitive performance, education, age, gender, and social activities served as moderators. In the following, we report significant and marginally significant interactions.

**FIGURE 3:**
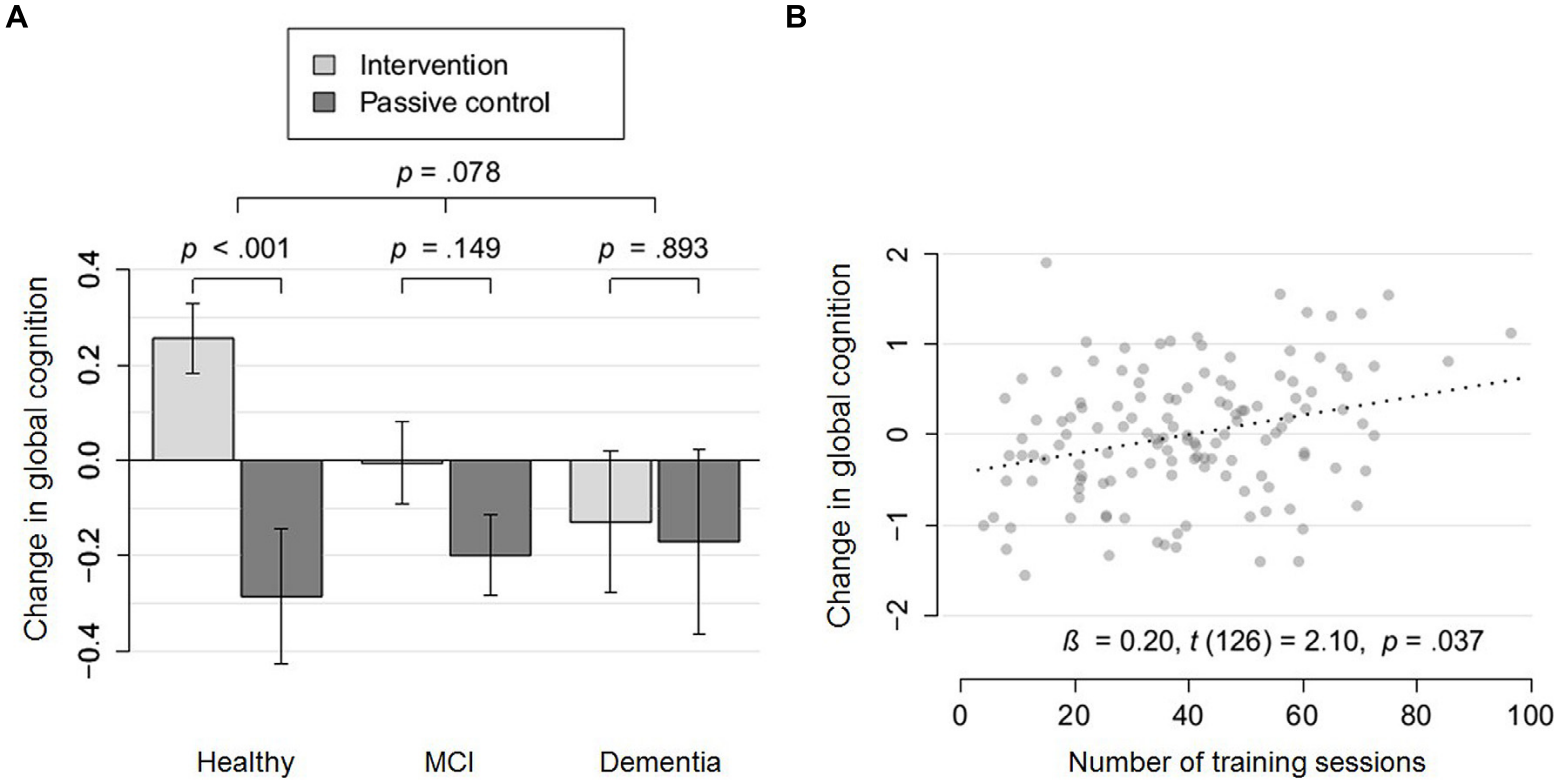
**Moderation and dose-response effects for global cognition. (A)** Change in global cognition (partial residuals accounting for covariates) of the intervention group (light gray), compared to the passive control group (dark gray) within cognitively healthy participants, participants with mild cognitive impairment (MCI) and dementia. Arrows represent SE. **(B)** Change in global cognition (partial residuals accounting for covariates) as a function of the number of training sessions within a subsample which were either cognitively healthy or diagnosed with mild cognitive impairment.

Regarding change in global cognition, the interaction term Group × Severity of NCD proved marginally significant, *t*(217) = 1.77, *p* = 0.08. With increasing severity of NCD, the intervention effect on global cognition decreased (see **Figure 3**). While healthy participants showed a highly significant intervention effect on change in global cognition, *t*(86) = 3.48, *p* = 0.0008, Cohen’s *d* = 0.54, participants with MCI, *t*(108) = 1.45, *p* = 0.15, Cohen’s *d* = 0.19, and dementia, *t*(25) = 0.14, *p* = 0.89, Cohen’s *d* = 0.04, did not show a significant improvement. It is of note, that according to the AIC, the model which accounted for severity of NCD as an effect modifier (AIC = 450.1) was preferred to the model which did not account for it (AIC = 455.5). However, taking multiple comparisons for the six moderators into account, the interaction effect would not remain significant, *p_adjusted_* = 0.47. The results indicate that this exploratory analysis is of use for the formulation of specific hypothesis which need to be tested more rigorously in future trials before clinical decisions can be based on them (Roback and Askins, 2005).

Regarding change in executive function, the interaction term Group × Baseline Executive Function proved significant, *t*(155) = 3.59, *p* = 0.0004. The lower the baseline executive function, the higher the intervention effect even after adjusting for multiple comparisons, *p_adjusted_* = 0.003. We also found significant moderator effects of age and severity of NCD which did not remain significant after adjusting for multiple comparisons, *t*(155) = 2.25, *p* = 0.03, *p_adjusted_* = 0.13, *t*(154) = 2.04, *p* = 0.04, *p_adjusted_* = 0.17, respectively. The younger participants and the more severe the NCD, the less improvements were induced in executive function. Importantly, if the interaction terms of all three moderators were included in one model, effects remained similar. The interactions Group × Baseline Executive Function, Group × NCD and Group × Age remained at least marginally significant, *t*(152) = 3.33, *p* = 0.001, *p_adjusted_* = 0.007, *t*(152) = 2.59, *p* = 0.01, *p_adjusted_* = 0.05 and, *t*(152) = 1.87, *p* = 0.06, *p_adjusted_* = 0.26, respectively. Lower baseline performance moderated the intervention effects among cognitively healthy participants, *t*(70) = 2.84, *p* = 0.006, *p_adjusted_* = 0.02, as well as, within participants with NCD, *t*(78) = 2.54, *p* = 0.01, *p_adjusted_* = 0.07, supporting the robustness of the moderator effect independent of severity of NCD. Education, gender, and social activity level showed no significant moderation effect, all unadjusted *p*s > 0.10.

In conclusion, regarding global cognition a tendency for a reduced intervention effect with more severe NCD was found. Regarding executive function, with higher baseline performance, more severe NCD and younger age, training-induced benefits were reduced.

### Does Training Dosage Matter?

To assess dose-related effects of training, we added the predictor training dosage (i.e., number of completed training sessions) to the regression model accounting for baseline cognitive performance, education, age and study center. For this analysis, we included only participants of the intervention group which started the intervention (*n* = 154). The number of training sessions marginally significantly predicted improvement in global cognition, β = 0.17, *t*(146) = 1.85, *p* = 0.07, and executive function, β = 0.23, *t*(103) = 1.92, *p* = 0.06 (see **Table 2**). With respect to episodic memory and working memory, no significant dose-response effect was found, *p*s > 0.356 (see **Table 2**).

**Table 2:** Effects of intervention group and dosage on change in cognition.

	Group^a^			Number of training sessions^a^		
Change in cognitive performance	Cohen’s *d*	*t* (df)	*p*-value	β ^b^	*t* (df)	*p*-value
Global cognition	0.31	3.20 (219)	0.002	0.17	1.85 (146)	0.07
Executive function	0.37	2.56 (156)	0.01	0.23	1.92 (103)	0.06
Working memory	0.15	1.29 (219)	0.20	0.10	0.93 (146)	0.36
Episodic memory	0.20	2.21 (216)	0.03	-0.01	0.13 (145)	0.90

*^a^All regression models accounted for baseline cognitive performance, education, age, and trial site.*

*^b^Standardized coefficient: it is predicted that every 18 sessions global cognition and executive function improves by 0.17 and 0.23 SD, respectively.*

Taking the moderator effect of NCD on global cognition into account (see **Figure 3A**), we conducted a dose-response analysis in the subgroup of non-demented participants (either cognitively healthy or diagnosed with MCI; *n* = 131). A significant dose-response effect was revealed for this subsample, β = 0.20, *t*(126) = 2.10, *p* = 0.04. Taking the robust moderator effect of baseline performance on executive function into account, we conducted a dose-response analysis for participants with low baseline executive function (median split; *n* = 56). We found a highly significant dose-response effect for this subsample, β = 0.54, *t*(51) = 2.83, *p* = 0.007.

The manipulation check was successful as we found a high correlation between the number of completed training sessions and the number of potential training sessions (*r* = 0.74, *p* < 0.001). Importantly, not only the completed training sessions but also the number of potential training sessions significantly predicted improvement in global cognition both within all participants of the intervention group, β = 0.20, *t*(151) = 2.37, *p* = 0.02, and within non-demented participants in the intervention group, β = 0.23, *t*(131) = 2.69, *p* = 0.008.

### Does Training Improve Secondary Physical, Psychological, and Daily Life Outcomes?

In a subset of study participants we assessed physical fitness and tested whether manipulation was successful. The intervention group compared to the control group significantly improved in physical fitness, *t*(117) = 6.50, *p* < 0.001 (see Supplementary Table S1). Psychological and daily life outcomes did not benefit from the intervention even without adjusting for multiple comparisons, *p*s > 0.09 (see Supplementary Table S1).

## Discussion

Mono-therapeutic interventions of physical and cognitive training have shown task- and domain specific cognitive benefits, but limited generalization effects on global cognition (Owen et al., 2010; Smith et al., 2010b; Kelly et al., 2014b; Rebok et al., 2014), especially in older adults (Schmiedek et al., 2010; but see also Lampit et al., 2014a). Our results indicate that combining physical and cognitive training can overcome this shortcoming. In a community-dwelling sample of cognitively healthy and impaired older adults, we provide evidence that intensive short-term physical and cognitive training induced benefits in global cognition (Cohen’s *d* = 0.31), executive function (more specifically switching, Cohen’s *d* = 0.37) and episodic memory (Cohen’s *d* = 0.20). Working memory improvement was not statistically significant (Cohen’s *d* = 0.15).

In addition, we found evidence for effect modifiers of cognitive gains in an exploratory approach. Regarding global cognition, a tendency for reduced intervention effects with more severe NCD was revealed. Regarding executive function, we found a robust moderation effect of baseline performance. The lower the baseline performance, the more benefits were found. We also found that participants with more severe NCD (healthy < MCI < dementia) and younger in age benefited less in executive function.

Consistent with the intervention effects on global cognition and executive function, we found evidence for dose-response effects within the subsamples which benefited most from the intervention. For individuals without dementia, the more training sessions were completed, the more benefits in global cognition were found. For individuals with low baseline executive function (<median), the more training sessions were completed, the more gains in executive function were revealed. These dose-response effects strengthen the interpretation that the cognitive benefits are attributable to the training components rather than unspecific characteristics of the intervention (cf. Hill, 1965).

Is the effect size of practical significance? According to the dose-response analysis global cognition is predicted to increase by 0.9 SD after 100 training sessions. In our sample, healthy adults were 0.56 SD better in global cognition than participants with MCI which were, in turn, 0.61 SD better than participants with dementia. Hence, the expected effect size of 100 training sessions is larger than the progression from healthy to MCI and from MCI to dementia.

### Group and Dose-Response Effects on Global Cognition and Specific Cognitive Functions

To our knowledge, this is the first study which showed combined training-induced improvement in global cognition of older adults within both a control group comparison and a dose-response analysis. The global improvement of cognitive performance is probably induced by multiple additive and interacting mechanisms of physical and cognitive training. One central mechanism of transfer effects may be the cognitive training-induced reorganization of neuronal networks enabling more efficient perceptual (Berry et al., 2010) and executive processing (Subramaniam et al., 2014). Transfer effects may be mediated via overlapping processing demands of cognitive tests and cognitive training (Dahlin et al., 2008). Possibly, the brain’s reorganization by cognitive training may have been potentiated by physical training-induced “plasticity facilitation” (Fissler et al., 2013).

Importantly, the transfer tasks we used to assess global cognition were structurally rather dissimilar from the cognitive training tasks (cf. Rebok et al., 2014). Therefore, it is more likely that transfer effects are not induced by strategy use or task-specific skills but rather by broad cognitive benefits in different domains. At the first glance, non-significant working memory effects seem inconsistent with other working memory studies (Karbach and Verhaeghen, 2014). However, highly consistent with the current literature, a subgroup analysis indicated medium-sized near-transfer effects on verbal working memory in cognitively healthy participants (Smith et al., 2009; Zelinski et al., 2011; Karbach and Verhaeghen, 2014), but non-significant effects in participants with NCD (Barnes et al., 2009, 2013). Interestingly, the TMT, which showed the largest effect sizes in the group and dose-response analysis, showed the lowest structural similarities with the cognitive training tasks indicating rather broad cognitive improvements by combined cognitive and physical training.

### Individual Differences in Training-Induced Benefits

The mechanisms of the moderation effect of severity of NCD, baseline performance, and age on training-induced cognitive benefits are speculative but may be explained via training-induced improvement in neurofunctional efficiency (Subramaniam et al., 2014). Participants with more severe NCD may have a reduced structural brain capacity (such as reduced number of neurons, synapses, and level of dendritic arborization; Arnold et al., 2013) limiting structural resources necessary for training-induced gains in processing efficiency (i.e., more efficient brain connectivity; Frantzidis et al., 2014). Participants with lower baseline executive function may have a reduced baseline processing efficiency which enables a larger zone of potential improvement. Older participants may have increased baseline variation in processing efficiency (Raz et al., 2005), which on average, increases the zone of potential improvement.

Recent studies support the finding of reduced effects in participants with NCD. Smith et al. (2009) and Zelinski et al. (2011) used the English version of this study’s cognitive training program and found improvements on verbal memory in a healthy sample. In other studies investigating participants with probable MCI, no significant effects of this program were found (Barnes et al., 2009, 2013). Applying a 6-months cognitive intervention, Buschert et al. (2011) found cognitive gains in participants with MCI but not among individuals with mild Alzheimer’s disease. In addition, recent meta-analyses on cognitive training revealed no cognitive benefits in participants with dementia while cognitive improvement was found in healthy older adults (Bahar-Fuchs et al., 2013; Karr et al., 2014; Kelly et al., 2014a). However, none of these studies investigated effect-modifying effects of severity of NCD through analyzing the Group × Severity of NCD interaction which is essential for conclusions. Thus, this study provides preliminary evidence for effect modification which should be further assessed in future long-term trials. It is important to note that reduced benefits for participants with more severe NCD may be a spurious finding because of an increased risk of false positives in an exploratory analysis. Furthermore, effect-modifying effects may be specific for certain training types or may not be found with more prolonged training (cf. Sitzer et al., 2006; Buschert et al., 2010). A prolonged increase in challenging activities might not primarily act on the reorganization of neuronal networks to increase processing efficiency but by disease-modifying mechanisms such as reductions in Aβ-deposition (Lazarov et al., 2005; Liang et al., 2010; Landau et al., 2012), prevention of synaptic loss (Arnold et al., 2013), neuronal death (Valenzuela et al., 2011), hippocampal atrophy (Valenzuela et al., 2008; Erickson et al., 2011; Smith et al., 2014), and whole-brain atrophy (Mortimer et al., 2012). Indeed, a recent study revealed clinically significant long-term effects of prolonged engagement in cognitively and physically challenging leisure activities such as gaming and Tai Chi on cognitive decline in a sample of older persons with dementia (Cheng et al., 2013).

Enhanced training-induced cognitive gain in participants with low baseline performance is consistent with findings from other cognitive and physical training studies (Mahncke et al., 2006b; Peretz et al., 2011; Barnes et al., 2013), game-based cognitive interventions (Whitlock et al., 2012; Baniqued et al., 2014), and a multimodal dancing intervention (Kattenstroth et al., 2013). All of these studies found increased cognitive benefits with lower baseline performance.

### Limitations

Blinding of test administrators and participants, as well as random allocation to intervention groups and training dosage was not feasible due to logistic and practical issues and time and financial limitations of the project, as discussed above. In addition, the use of a passive control group cannot exclude motivation or expectation-based intervention effects such as Hawthorne and placebo effects. However, the lack of randomization is unlikely to bias effects as baseline characteristics between the groups are comparable. In addition, consistent training-induced cognitive benefits in both the group analysis and the dose-response analysis make a bias in favor of intervention-induced cognitive benefits unlikely. Furthermore, participants were blind with regards to the different training dosages which make Hawthorne and placebo effects less likely. In addition, Hawthorne and placebo effects are unlikely to explain medium-sized global cognitive benefits as previous large-scale studies and meta-analysis demonstrated no differences between active and passive control groups (Ball et al., 2002; Kelly et al., 2014b; Lampit et al., 2014b; Park et al., 2014).

The number of completed training sessions was not fully explained by the manipulation of the number of potential training sessions (*r* = 0.74). Thus other variables related to the number of completed training sessions such as participants’ motivation or time limitation might have contributed to the dose-response effect. However, this explanation is unlikely as not only the number of completed training sessions but also the manipulated number of available training sessions showed a beneficial effect on global cognition.

Outcome measures for the assessment of global cognition were limited to three cognitive tests of three cognitive functions. Thus, we do not know whether the measure of global cognition would have improved if more cognitive tests measuring more cognitive functions would have been included. However, with our three measures of executive function (switching), working memory and episodic memory, a wide spectrum of fronto-parietal and mediotemporal lobe functions – most affected in aging - were assessed (Park et al., 2002; Raz and Rodrigue, 2006; Bamidis et al., 2014). Finally, the TMT could not be conducted within time limits by 29% of the intervention group and 26% of the control group. As missing data did not differ between groups, we do not expect that it biased effects.

Due to a lack of studies investigating effect modifiers of combined physical and cognitive training, an exploratory approach with multiple comparisons was necessary. This approach increases the risk for false positives or - if Type I error is adjusted – increases the risk for false negatives (Roback and Askins, 2005). To our knowledge, this is the largest study which assesses an effect-modifying effect of severity of NCD revealing small- to medium-sized differences between cognitive benefits of cognitively healthy participants (*d* = 0.54), participants with MCI (*d* = 0.19) and dementia (*d* = 0.04). These effect sizes are of clinical significance, but not of statistical significance after adjusting for multiple comparisons. Hence, the trend for severity of NCD as an effect modifier in the unadjusted analysis should be used to justify rigorous hypothesis testing in future trials but, yet, not for clinical decisions (Roback and Askins, 2005).

### Future Directions

Future studies should extend our results of combined cognitive and physical training by investigating other outcome measures and maintenance of effects. Sensitive and objective measures of daily functioning should be used (Tucker-Drob, 2011) to better understand the significance of cognitive improvements for daily life. Effects of combined training on molecular (neurotrophins, amyloid deposition, metabolomic and lipidomic biomarkers) and neuronal correlates of cognition (structural and functional brain networks) should be investigated to reveal the underlying mechanisms of effects (see Bamidis et al., 2014 for a review). More long-term follow-up studies need to be conducted in order to reveal maintenance of effects (Rebok et al., 2014). Most importantly, large-scale studies with longer training duration need to be conducted to investigate the effect of combined training on the long-term incidence and trajectory of NCD in relation to NCD severity (cf. Unverzagt et al., 2012). These important, but unexplored outcomes of combined physical and cognitive training should be investigated within randomized controlled trials, the gold standard to accurately estimate the true effect of interventions because of their ability to minimize bias (Moher et al., 2010).

Effects of combined physical and cognitive training need to be decomposed to better understand the contribution of each component and their synergy (see Fissler et al., 2013 for a review). Decomposing of effects while keeping training time constant can be established by comparing simultaneous physical and cognitive training vs. individual components (Anderson-Hanley et al., 2012) or by a 2 × 2 factorial design with placebo control conditions (Barnes et al., 2013). Temporal proximity and the sequence of combined training types (i.e., physical before cognitive training or vice versa) should be manipulated systematically. Temporal proximity and sequence may be decisive for a synergy effect of cognitive and physical training as training-induced neurotrophin up-regulation peaks after about 2 h and declines to baseline level afterward (Rasmussen et al., 2009).

## Conclusion

Neurocognitive disorders and brain pathology are insidious phenomena which begin decades before their diagnosis (Braak et al., 2011). Hence, strategies for the prevention of dementia must start long before neurocognitive deterioration impairs activities of daily living. Here, we provide evidence that combined training induces dose-responsive improvement in global cognition, especially in individuals with less severe NCDs. Whether effects on global cognition through combined training may reduce the incidence and the trajectory of NCDs in relation to its severity must be assessed in future long-term randomized controlled trials.

## Author Contributions

PB coordinated the whole project and study in all study centers for the duration of all project phases, conceptualized the integrated software system and the physical training system for the intervention, supervised subject recruitment, data acquisition and analysis, interpreted the data, wrote parts of the manuscript and revised the manuscript critically for important intellectual content. PF wrote the manuscript, supervised the data acquisition, performed the data analysis and interpreted the data. SP contributed to the conceptualization and study design, worked on the data acquisition (especially in recruiting and diagnosing participants), supervised the neurological and neuropsychological assessments, and critically revised the manuscript for important intellectual content. VZ and ER worked in the localization of the training software, the conduction of the trials, the data acquisition, data entry and data filtering, and performed parts of the initial data analysis and revised the manuscript for important trial and data-related content. EK and AB designed and implemented the physical training software, technically supported all trials during the whole duration of the project, were involved in data acquisition and data interpretation and revised the manuscript for important technical and intervention related content. MK, IB, AT, GT, EG, AL, AK, ES, EM, and AN were responsible for subject recruitment, the neuropsychological assessment and conduction of the trials, the data acquisition, data entry, performed parts of the initial data analysis and revised the manuscript for important process-related content. AT, MT, SM, and JP were responsible for subject recruitment, the neurological assessments and the subject diagnosis, data acquisition, data entry, and critically revised the manuscript for important methodology-related content. CF and AS were responsible for the coordination of trial center settings, subject recruitment, the neurophysiological recordings and assessment, the data acquisition and filtering, data entry, performed parts of the initial data analysis and revised the manuscript for important trial and data-related content. WS and TE were involved in the design of the study, monitored all trials for quality assurance, supervised statistical analyses, and revised the manuscript critically for important intellectual content. AV contributed to the design of the study, conceptualized part of the trial software, supervised the neuropsychological assessment and data collection, contributed substantially to the data analysis and interpretation, and revised the manuscript critically for important intellectual content. I-TK conceptualized the study, conducted important pre-experiments and worked in the localization of the Brain Fitness Program, supervised statistical analyses, and revised the manuscript critically for important intellectual content. All authors gave their final approval to the version to be published and agreed to be accountable for all aspects of the work in ensuring that questions related to the accuracy or integrity of any part of the work are appropriately investigated and resolved.

## Conflict of Interest Statement

Iris-Tatjana Kolassa and Thomas Elbert are members of the scientific advisory board of Posit Science. The other authors declare that the research was conducted in the absence of any commercial or financial relationships that could be construed as a potential conflict of interest.
