# Supplementary material for: Gains in cognition through combined cognitive and physical training: the role of training dosage and severity of neurocognitive disorder
Source: Front Aging Neurosci. 2015 Aug 7;7:152. doi: 10.3389/fnagi.2015.00152 (PMC4528297; doi:10.3389/fnagi.2015.00152)
Supplement: Supplementary file 1 [file Table_1.PDF]

Supplementary Material

Gains in cognition through combined cognitive and physical training: dosage and severity of neurocognitive disorder matters

Panagiotis D. Bamidis[1,a*], Patrick Fissler[2,a], Sokratis G. Papageorgiou[3,a], Vasiliki Zilidou[1], Evdokimos I. Konstantinidis[1], Antonis S. Billis[1], Evangelia Romanopoulou[1], Maria Karagianni[1,4], Ion Bearatis[3], Angeliki Tsapanou[3], Georgia Tsilikopoulou[3], Eirini Grigoriadou[1,4], Aristea Ladas[1,5], Athina Kyrillidou[1,4], Anthoula Tsolaki[1,4], Christos Frantzidis[1], Efstathios Sidiropoulos[1], Anastasios Siountas[1], Stavroula Matsi[3], John Papatriantafyllou[3], Eleni Margioti[3], Aspasia Nika[3], Winfried Schlee[6], Thomas Elbert[7], Madga Tsolaki[4,8], Ana B. Vivas[5,b], Iris-Tatjana Kolassa[2,b]

[1] Laboratory of Medical Physics, Faculty of Health Sciences, Medical School, Aristotle University of Thessaloniki, Thessaloniki, Greece
[2] Institute of Psychology and Pedagogy, Clinical & Biological Psychology, University of Ulm, Ulm, Germany
[3] Behavioral Neurology and Neuroposychology Unit, 1st and 2nd Department of Neurology, Medical School, National Kapodistrian University of Athens, Athens, Greece
[4] Greek Association of Alzheimer's Disease and Related Disorders, Thessaloniki, Greece
[5] Cognitive Psychology and Neuropsychology Lab, Department of Psychology, The University of Sheffield International Faculty, City College, Thessaloniki, Greece
[6] Department of Psychiatry and Psychotherapy, University of Regensburg, Regensburg, Germany
[7] Clinical Psychology and Clinical Neuropsychology, University of Konstanz, Konstanz, Germany
[8] 3rd Department of Neurology, Medical School, Aristotle University of Thessaloniki, Greece

[a] joint first authorship: Panagiotis D. Bamidis, Patrick Fissler & Sokratis G. Papageorgiou contributed equally and are joint first authors
[b] joint senior authorship: Ana Vivas & Iris-Tatjana Kolassa

*** Correspondence:** Panagiotis D. Bamidis, Laboratory of Medical Physics, Faculty of Health Sciences, Medical School, Aristotle University of Thessaloniki, PO Box 376, Thessaloniki, 54124, Greece.
bamidis@med.auth.gr

1. Supplementary Data

Table S1. Intervention Effects on Cognitive Subscore and Psychological, Physical and Daily Living Outcomes

	Intervention group (n = 163)				Control group (*n* = 66)				Group effect[a]
	Pre		Post		Pre		Post		
Measure	*M*	(*SE*)	*M*	(*SE*)	*M*	(*SE*)	*M*	(*SE*)	*p* Value
Global cognition	.03	(.08)	.42	(.09)	-.08	(.12)	.08	(.13)	.02
Executive function[b]	-.06	(.10)	.16	(.08)	.14	(.13)	-.10	(.17)	.01
Working memory	.04	(.08)	.28	(.10)	-.10	(.11)	.08	(.12)	.64
Episodic memory	.08	(.08)	.49	(.09)	-.20	(.12)	.09	(.14)	.17
MMSE	26.82	(.22)	27.25	(.20)	26.38	(.35)	26.80	(.36)	.99
TMT A[c]	79.9	(3.21)	71.5	(2.90)	82.2	(4.79)	73.4	(4.58)	.91
TMT B[c]	181.9	(6.61)	164.3	(6.48)	177.8	(9.87)	176.9	(9.96)	.03
Digit Span Forward	6.31	(.16)	6.55	(.16)	6.36	(.20)	6.38	(.22)	.29
Digit Span Backward	4.45	(.11)	4.84	(.15)	4.06	(.18)	4.50	(.16)	.86
VLT - Trial 1	5.29	(.17)	6.35	(.22)	5.09	(.27)	5.91	(.36)	.54
VLT - Trial 5	10.08	(.25)	11.17	(.27)	9.57	(.39)	10.05	(.43)	.07
VLT - Learning	41.15	(1.00)	46.75	(1.16)	38.20	(1.46)	42.40	(1.88)	.27
VLT - Delayed recall	8.20	(.34)	9.59	(.35)	6.94	(.53)	7.85	(.55)	.27
GDS-short[b]	2.88	(.23)	2.56	(.21)	2.02	(.25)	1.92	(.28)	.49
WHOQOL – Global	-.10	(.10)	.11	(.09)	.35	(.15)	.37	(.11)	.21
WHOQOL – Physical	25.42	(.37)	25.83	(.37)	26.82	(.66)	27.52	(.57)	.68
WHOQOL – Psychological	20.61	(.33)	21.18	(.33)	21.52	(.40)	21.55	(.36)	.29
WHOQOL – Social	9.22	(.20)	9.55	(.17)	10.69	(.40)	10.28	(.37)	.09
WHOQOL – Environmental	28.91	(.41)	29.78	(.35)	29.30	(.51)	29.52	(.44)	.36
Physical fitness	-.75	(.39)	2.51	(.44)	1.37	(.62)	1.59	(.66)	<.001
Chair stand	12.47	(.32)	14.88	(.42)	13.51	(.56)	13.23	(.58)	<.001
Arm curl	16.05	(.45)	20.78	(.53)	19.97	(.66)	20.23	(.79)	<.001
2-minute step	86.44	(6.11)	103.5	(6.25)	92.74	(8.98)	105.7	(13.5)	.48
Back scratch	-10.3	(1.11)	-7.53	(1.06)	-11.3	(1.90)	-11.1	(1.98)	.002
Chair sit-and-reach	-1.27	(1.14)	2.93	(1.18)	.14	(1.35)	-.20	(1.27)	<.001
8-foot up-and-go[c]	6.50	(.17)	5.74	(.16)	5.55	(.26)	5.47	(.22)	.002
IADL (only women[d])	7.76	(.09)	7.76	(.09)	7.70	(.15)	7.68	(.14)	.69

[a] Group effect without accounting for covariates

[b] If participants could not complete the Trail Making Test within time, the Executive function score could not be calculated: 29% and 26% of scores were missing in the intervention and control group, respectively. Differences between groups were not significant, $\chi^2(1) = 0.16$; $p = .69$.

[c] lower values indicate a more favorable outcome; if participants took longer than 180 seconds for TMT A and 300 seconds for TMT B, they received the respective maximum score

[d] This measure was analyzed only in women as men commonly engaged in only 5 out of 8 activities which was not due to inability to perform activities

WHOQOL = World Health Organization's Quality of Life Questionnaire; MMSE = Mini Mental State Examination; GDS = Geriatric Depression Scale; IADL = instrumental activities of daily living; TMT = Trail Making Test
